# Groups' Actions Trump Injunctive Reaction in an Incidental Observation by Young Children

**DOI:** 10.1371/journal.pone.0107375

**Published:** 2014-09-08

**Authors:** Cameron R. Turner, Mark Nielsen, Emma Collier-Baker

**Affiliations:** 1 School of Education, Durham University, Durham, United Kingdom; 2 Early Cognitive Development Centre, School of Psychology, The University of Queensland, Brisbane, Australia; 3 School of Applied Human Sciences, University of KwaZulu-Natal, Durban, South Africa; Birkbeck, University of London, United Kingdom

## Abstract

Children's ability to use social information to direct their behavior is key to their survival and development. However, in observing adult behavior, children are confronted with multiple forms of social information that may vary in reliability and adaptiveness. Two of the most well established biases influencing human behavior are: (1) following the majority (majority influence or conformity); and (2) the use of emotional signals. The current experiment aimed to evaluate how children respond when both information about the majority behavior of a group (descriptive norm) and attitudes of the group towards a behavior (injunctive norm, expressed through an emotional reaction) are present and what happens when they are in conflict. We used a method designed to mimic the manner in which children might observe group members' behavior during development. Novel apparatuses were constructed for which there were two discrete actions that could be performed to retrieve a reward. Three-year-olds observed four adults demonstrating one set of actions, followed by a fifth adult who presented an alternative set of actions. The first four adults' injunctive responses to this fifth adult's actions were manipulated between-groups: positive, negative, or neutral. It was found that children preferred to copy the majority action, regardless of the injunctive reaction of the group. We argue that this affirms the adaptive utility of copying the majority.

## Introduction

A considerable amount of human social behavior is governed by norms; with individuals both adhering to – and expecting others in their group to adhere to – certain common and agreed upon behaviors within given contexts. For instance, norms are crucial in both social relations (e.g., shaking hands upon meeting someone new), and in tool-use (e.g., using a knife in your right hand and fork in your left when eating). Social psychologists have long conceptualized norms as being able to be analyzed along two components: (1) the frequency with which a behavior is exhibited, and (2) the groups' approval of that behavior [1]. That is, norms are generally behaviors which most or all individuals do, and which most approve of doing. Norms are increasingly being identified as important in child development and culture [2], and there has been recent interest in examining children's preferential copying of majorities of individuals [3] [4] [5]. However, the drawing-in of norm-based theories from social psychology with adults into developmental psychology remains in its early stages. An important cleavage which has not yet been investigated in children is between descriptive norms (the behavior most individuals in a group actually do in a context), and injunctive norms (the behavior most individuals in the group think one should do in a context) [6]. This division between the most exhibited actions and the expressed attitudes of a group to those actions has been shown to help explain adult behavior (e.g., [6] [7] [8]). Although caregivers commonly instruct children on how to behave based on injunctive norms little is known about how children process such information and prioritize it relative to descriptive norms. Thus, following social psychological research with adults, the current experiment examined how the interaction of these two normative processes influences children's behavior. Given its likely importance in underpinning adults' injunctions witnessed by children during development, we begin by reviewing the role adults' emotional reactions play in children's social learning. We follow this by examining research into majority-biased copying and conformity in children as it reflects research into the effect of descriptive norms on children's behavior.

In his concluding remarks of *The Expression of the Emotions in Man and Animals* Darwin writes: “The young and the old of widely different races, both with man and animals, express the same state of mind by the same movements” ([9] p. 352). His conjecture was that we see in humans of all cultures common representations of emotions on the face, and that this proclivity to show emotion by specific behaviors was shared by non-human animals. Darwin believed emotional expressions were part of the inheritance of the organism, a product of evolution by natural selection.

Following Darwin, Shariff and Tracy [10] argue that emotional expressions have evolved to communicate information about our internal conditions, our emotional states. The first piece of evidence Shariff and Tracy bring to bear on this claim is Ekman and Izard's demonstration of the cross-cultural universality of the understanding of facial expressions [11] (for contrasting views see [12] [13]). Secondly, the human nervous system appears to be designed to respond rapidly to emotional expression, with subcortical loops recruited in response to fear expressions, capturing attention and allowing for detailed perceptual processing [14]. Furthermore, we see evolutionary preparedness in response to facial expressions. Fear and anger expressions are more easily paired with aversive stimuli than expressions of positivity – threat signals and actual threats condition together rapidly [15]. Emotional expression and recognition reliably develop in all non-disordered children in all cultures.

If emotional expressions have evolved to signal information, when do children first begin to interpret these expressions and use them to inform their behavior? Montague and Walker-Andrews [16] showed that 4-month-old infants could already decipher facial expressions (happiness, anger, sadness and fear), and, in a twin-study with 5-year-olds, Elam, Carlson, DiLalla and Reinke [17] showed that the ability to direct attention to faces had a significant genetic component. Congruently, research into infant social referencing has shown directly that children use the emotional reactions of adults to inform their own behavior. By 12 months infants can use emotional signals to regulate their actions [18], and are also able to interpret others' actions in light of the emotions they express [19].

Repacholi and Meltzoff [20] give perhaps the most persuasive demonstration of infants' use of adults' emotional signals in social learning. They showed that 18-month-olds ‘emotionally eavesdrop’: In other words, they learn from incidental observations of adult emotion, and adjust the behaviors they imitate accordingly. In Repacholi and Meltzoff's study, an adult performed an object-directed action (e.g., pulling a toy dumbbell apart) that was followed by another adult acting either neutrally or angrily towards the demonstrating adult. Children imitated the target action at lower levels if the target action had been previously met with anger – so long as the reacting adult could see what the child was doing. This built on prior research showing that infants will adjust their interaction with objects depending on the emotional reactions of a model using the object [21] [22] [23] [24] [25] [26], providing good evidence that children use the emotional expressions of adults to guide their own behavior. It has been further proposed that the negative emotional expressions of adults may be especially strong in influencing children's behavior because of the potentially higher evolutionary costs of the information they express [27].

We know from classic demonstrations [28] [29] that copying the majority is a further powerful psychological bias in humans. People have a strong preference to copy the behavior of others, even against the evidence of their own perception (for a review see [30]). This too is plausibly an evolutionarily prepared bias: copying others leads to liking, to social acceptance [31] [32]. However, it has also been demonstrated that copying others' behavior will tend to produce the optimal behavior in a given context, and therefore maximize fitness to the individual, under quite a staggering range of conditions [33] [34]. Many authors have suggested that much of the research into majority influence and conformity in both humans and animals is explicable in terms of these two motivations: increasing social acceptance and behaving in accord with reliable information [3] [4] [5] [35].

Recently, evidence has emerged that children also appear to be prolific in copying the majority. Haun and colleagues [4] draw attention to a distinction between instance of “majority influence” where individuals copy the most frequent behavior, and “conformity” where it is demonstrated that the individual is changing from their preference to adhere to the preference of the majority; we bear this distinction in mind while discussing the following literature. Corriveau and Harris [36] had three adult models all simultaneously incorrectly identify which of three lines matched a fourth, and found that three- and four-year olds made similar responses to Asch's adults: that is, they identified a clearly different line as matching the target. In an earlier study, Walker and Andrade [37] also employed an analogue of Asch's paradigm with adult models presenting incorrect judgments in serial, with 3 to 17 year olds exhibiting a strong tendency for conformity. Haun and Tomasello [38] found similar evidence for conformity in which children compared the size of drawings of animals. Corriveau, Kim, Song and Harris [39] in a line judgment task with 3- and 4-year-olds found that Asian-American children showed greater conformity to the majority than Caucasian-American children. Haun, Rekers, and Tomasello [40] demonstrated that both two-year-olds and chimpanzees (but not orangutans) will preferentially copy behaviors performed by a majority (demonstrating majority influence). That is, in order to receive a reward they will preferentially choose to drop balls into a box selected by three role models once over an alternative box chosen by one role model three times. Corriveau, Fusaro, and Harris [41] found young children preferred to endorse information given by an individual whose choices matched the majority, rather than a dissenter. And Seston and Kelemen [42] showed analogous effects within the object function domain: children preferring to match the object functions given by majority members demonstrating a consensus.

This research examining conformity and majority influence in children comes not only off the back of research with adults, but also from research showing children's profound proclivity to reproduce the actions of adults [5]. The most powerful demonstration of this proclivity comes from work on the overimitation effect [43] [44] [45]. In a landmark study, Horner and Whiten [46] modeled to both young children and chimpanzees a set of actions by which a reward could be retrieved from two identical apparatuses. The actions and the apparatuses were identical, except one apparatus was opaque and the other transparent. When the experimenter modeled the actions on the transparent apparatus it was apparent that some of the actions used were in fact redundant. Children and chimpanzees copied all of the demonstrated actions on the opaque box. However when presented with the transparent apparatus the chimpanzees jettisoned the redundant actions. In contrast, human children copied the sequence of actions with high fidelity across apparatuses. The overimitation effect has been shown to be robust [44], a likely human universal [47] [48], and performed at increasingly higher rates with age, even into adulthood [49] [50].

There has been much debate about the adaptive value of overimitation and children's penchant for high-fidelity copying in general. Complicating this debate is the finding that children will, under certain circumstances, ‘rationally’ imitate, omitting redundant actions when there is a logical reason to [51], yet in other cases will perform redundant actions even when instructed and incentivised not to [44]. This balance, however, appears to shift through the third year from an inclination to imitate rationally to an inclination to overimitate [52]. Over and Carpenter [5] have recently attempted to resolve this rational imitation-overimitation paradox by suggesting research into infant social learning be placed in the context of social psychological research into conformity/majority influence with adults. They argue that the same two biases underpin conformity/majority influence and imitation: (1) an interpersonal function increasing liking and affiliation [53], and (2) an accuracy function, facilitating learning about what are the most effective behaviors in a given situation. This mirrors earlier claims by Uzgiris [54] about the dual roles of imitation and the motivations underpinning conformity/majority influence by Claidière and Whiten [3], and others [5] [35]. Over and Carpenter [5] suggest that in both majority influence/conformity research and in overimitaiton/imitation research individuals are copying not just to gain useful information but to transmit the message “we are alike”. Over and Carpenter [5] further argue that overimitation, and differences between contexts when children overimitate, can be explained by reference to: (1) the child's own goals (to learn or to increase social affiliation) in the situation; (2) the child's social identification with the model; and (3) the social pressures of the imitative situation. In this way research into imitation and overimitation can be seen as a demonstration of a powerful bias in children for copying the behavior of adults, which is on a continuum with copying the behavior of a group of adults – what is more traditionally labeled as ‘conformity’ or majority influence.

We see in children a strong bias for copying the behavior of a majority of adults as well as a strong bias for basing behavior on adults' emotional and injunctive reactions towards behaviors. Within social psychological research conducted with adults, how the group perceives actions as *wrong* or *right*, and the behaviors the majority performs have both been thought to be important in influencing the actions group members adopt [35]. In the theory of planned behavior [55], the subjective norm – the amount of social pressure perceived by the individual to perform a behavior – was postulated to be a powerful force in influencing attitudes and behavior. Yet, due to repeated demonstrations of the weakness of subjective norms in driving behavior [56], this was reconceptualized into two distinct constructs: the *descriptive* norm and the *injunctive* norm. The descriptive norm refers to behavior most people in a group perform, whereas the injunctive norm refers to the behaviors most people in a group feel should be performed: That is, the *right* way to act. For instance, the injunctive norm might be that littering is *wrong* and that you should throw your rubbish in the bin; however, the descriptive norm may be that in fact most people in a given context (e.g., in a movie theatre) litter. There is evidence that in adults a preference to adhere to injunctive norms over descriptive norms [57].

The current experiment was designed to examine how both the bias to copy what most do, and what most think one should do, may differentially influence children's imitative choices. During development, children can be exposed to both sources of information: the injunctive reactions of their group members towards behavior (most saliently underpinned by emotional reactions), and the behavior most people are using. We wanted to know whether one source of information is privileged over the other. In the current experiment we thus set up opposing hypotheses to examine the relative influence of these biases.

**Figure 1:**
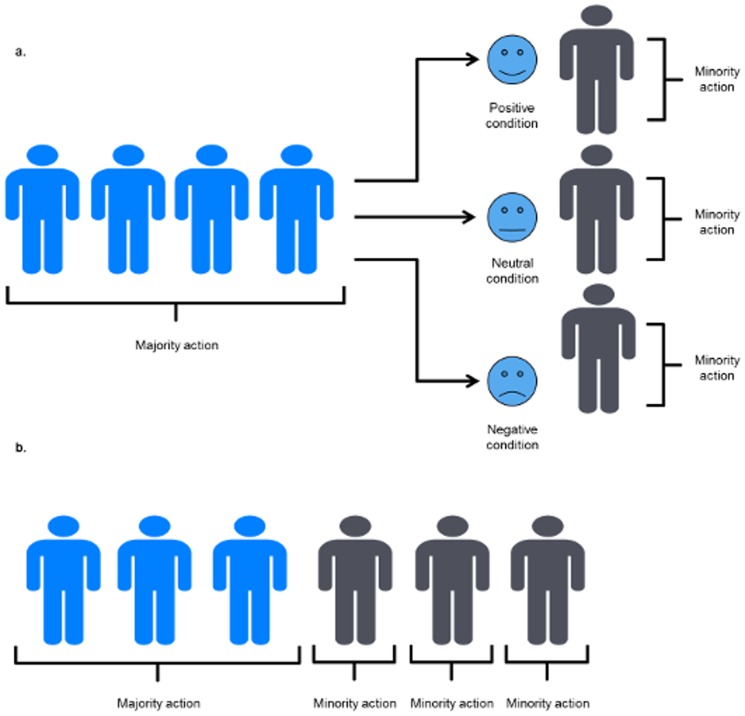
Schematic of the conditions within the experiment. (a) Experimental condition: after seeing a majority of four performing an action, saw a fifth actor performing a different action. The injunctive reaction of the group to this action varied between-subjects, being either positive, neutral or negative. (b) Frequency control condition: children saw a majority of three individuals performing a set of action, followed by three repetitions of the minority actions by a single individual.

Here children were exposed to apparatuses for which there were two discrete ways of retrieving a toy reward. Children observed four models opening the apparatus with one method, then a fifth model opening the apparatus with an alternative method. To create an injunctive norm towards a behavior, the emotional reaction, gesture and comments, of the first four models to the fifth model's action was varied: being either positive, negative or neutral. This design allowed us to compare the effect of majority influence with the effects of the normative injunctive reaction of a group towards a behavior (see Figure 1). Specifically, this design allowed us to look at how the groups' injunctive reaction towards a behavior may influence social learning more than just their performance of that behavior. It was also selected to attempt to mirror the social learning experience children go through in development, where they may see group members performing a behavior, but also violations of this normative behavior, which carry disapproval or approbation by the group. If children's imitation is more strongly influenced by the injunctive norm we would expect (a) variation in imitation based on the valence of the group. Namely, that children would imitate the lone individual model's behavior in accord with the groups' injunctive reaction: increasing their imitation of the lone-models' behavior in the positive condition relative to their imitation of her behavior in the negative condition. Conversely, if a copy the majority or ‘descriptive norm’ bias predominates, then we should expect (b) to find children preferring to copy the behavior used by the group at a significantly higher rate than the behavior modeled by the individual; with a preference for the descriptive norm over the injunctive norm would be shown if this effect extended across injunctive valence conditions. We also included a control condition in which the number of demonstrations were the same for both group and individual, which affords insight into the influence of demonstration frequency.

## Materials and Methods

### Ethics Statement

Research conducted after approval and under the supervision of The University of Queensland, adhering to Australian and International standards for conducting research with humans. Written consent was attained from parents/guardians/next of kin/caretakers for the participation of their child, who was also present throughout all testing.

### Participants

Fifty-two children participated in this research. Eight children were omitted from analyses due to parental interference (e.g., providing explicit instruction to the child on what to do), thus the final sample contained 25 boys and 19 girls, aged between 2.83 and 3.34 years (*M* = 3.08, *SD* = .13; *N* = 44, *n* = 11 per condition). Two- to three-year-old children were selected as this age group has generally been the youngest tested in comparable previous experiments (e.g. [36]). Children were recruited through a database of parents who had previously indicated willingness to have their children participate in research.

### Materials

**Figure 2:**
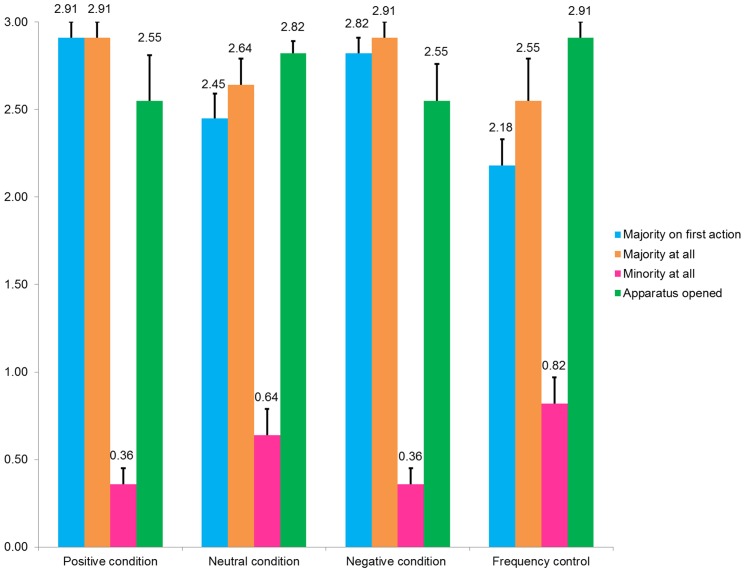
The three apparatuses used, descriptions of both sets of actions used to retrieve the reward toy, and description of the reward toys.

Children in each condition viewed demonstrations on three novel apparatuses, each of which had two discrete methods to retrieve a toy (see Figure 2). The order of presentation of these apparatuses was counterbalanced across participants. Using a range of apparatuses requiring distinct action sequences and for which the reward toys were different, removed the possibility that the results found could be attributed to the properties of any one apparatus. The adult models (2 male, 3 female) were presented via DVD on a large color flat-panel television.

### Procedure

To allow coding of behavior the children were videotaped. In establishing the preferred action of the group, and the group members' injunctive reaction towards an alternate behavior, all children were exposed to a series of videos of adults performing actions on the novel apparatuses. Videos began with the five models standing behind a table, where the first apparatus was placed. Going from right to left, the first four models stepped forward individually, opened the apparatus and, one after another, extracted the toy using the majority action. The fifth model then stepped forward and extracted the toy from the apparatus using the alternative action sequence. Models' responses to retrieving the toy were kept constant: All looked happy to retrieve the toy, briefly playing with it, then holding it towards the camera to make it salient. The reaction of the group to the lone model's action varied between-subjects by condition as described below. The video of the lone model's actions and the response of the group was played twice – thus in the injunctive reaction conditions there was one presentation of each of the four group members using one action sequence versus two presentations of the lone model's action sequence. This approach was taken in order to emphasize the group behavior as the majority approach. Nonetheless, to account for the possibility that children's use of the group action may not be due to conformity but rather to greater number of presentations, we also included a control condition in which the first three group members were shown performing the majority action followed by three repetitions of the lone model's action sequence (i.e., there were 3 presentations of each of the majority and minority actions).

Given previous literature revealing a strong bias in children to copy adults, especially in groups [40] [41], we wanted to provide an added incentive for children to copy the behavior of the lone model. Thus the two actions used to extract the reward toy varied. The action used by the group took longer to complete and incorporated causally inefficient actions, and the action used by the single member was quicker and more causally efficient (across the apparatuses, the former taking an average of 3.6 times longer for the adults model to achieve).

Before testing began, children were settled in a playroom. When children appeared relaxed, they, along with their parent and experimenter, entered the testing room. Children sat in front of the television screen, within arm's reach of their parents. They were directed to look at (but not touch) the first apparatus, which was placed on a table behind them. Children's attention was drawn to the apparatus prior to the demonstrations being presented in order to reinforce the value of watching the television. The experimenter said: “Alright you see what we have on the table here… Well we're going to watch a video, and some people are going to show you how they use it. Let's watch!” After viewing, children were presented with the first apparatus on the floor, the experimenter saying: “Ok, now you can have a turn, you can play with it however you like!” After an action on the first apparatus had been performed, it was removed, children were re-seated and the same protocol was followed with the remaining two apparatuses. Children were randomly allocated to one of the following four conditions:

#### Positive response condition

As the fifth (lone) model retrieved the toy, the four observers, each of whom had used the same approach, smiled, nodded and offered approving vocalizations (e.g., “mmm…”). One member of the group also stepped forward, saying: “Yes, that's a great way to do it!”, and gave a positive ‘thumbs-up’ gesture.

#### Negative response condition

This condition was identical to the positive response condition except the group members reacted to the lone model's retrieval method by frowning, shaking their heads, and making disapproving vocalizations (e.g., “tsk, tsk”). The same group member who individually responded in the *Positive* condition responded here by stepping forward, saying: “No, that's not how you do it!” and throwing his hands down in a negative gesture.

#### Neutral response condition

This condition was run as per the positive and negative response conditions except the group made no response to the new action, maintained neutral expressions throughout, and no member of the group stepped forward to comment on the lone model's action.

#### Frequency of presentation control

This condition was the same as the *Neutral* response condition except the child was shown three members of the group modeling the target actions (instead of four members) and three repetitions of the lone individual's demonstration (instead of two).

The use of videotaped images of adults expressing emotion [58], and this type of expression of emotion, gesture and language to manipulate adult opinions of behavior [58] [59], have been shown to be effective in previous social referencing research.

### Coding

For all three apparatuses in each condition the following was coded dichotomously.

- Whether the first action children attempted was that of the majority (1) or lone model (2).
- If they changed their action from the majority to the lone model's action: yes (1), or no (2).
- If they change from the lone model's action the majority's action: yes (1), or no (2)
- If they were successful in retrieving the toy: yes (1), or no (2).
- If the child used the groups' action at all during the trial: yes (1) or no (2).
- If they used lone model's action at all during the trial: yes (1) or no (2).

### Reliability

A second coder, blind to condition, recoded a random 30% of the videos. There was a high level of agreement between raters on each of the imitation task variables: *k* = 1.00, *p*<.001 for each measure. Due to the high level of agreement the original coder's data were used for analyses.

## Results

Measures were produced for key outcome variables by compiling the binary results for each trial over three trials for each child, giving a score out of three. Preliminary analyses failed to reveal any effect of apparatus type or apparatus order (these variables are not considered further), or effects of gender. Given the relatively restricted range of scores all analyses were conducted using parametric and non-parametric statistics. As these yielded the same outcomes, for ease of communication, only parametric statistics are reported here.

**Figure 3:**
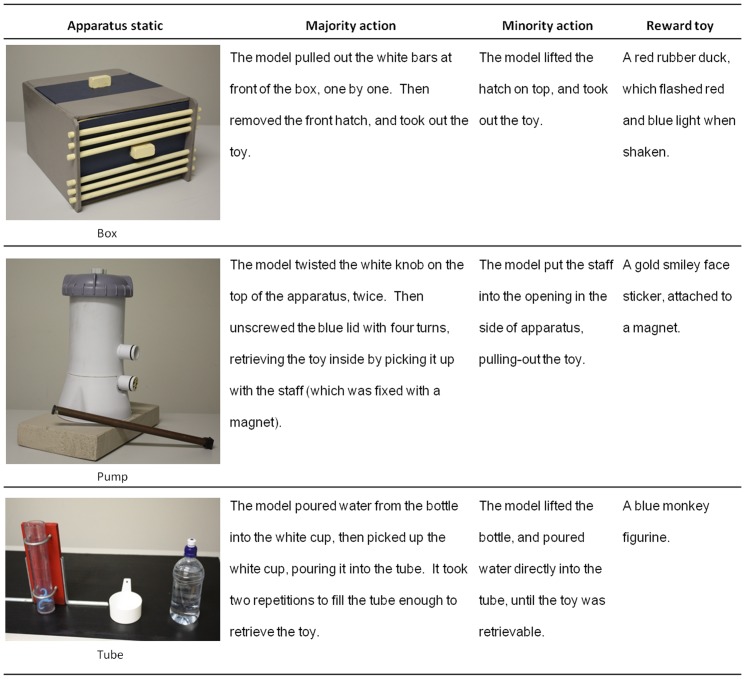
Means (and standard errors) across conditions for the number of apparatuses on key outcome measures.

As can be seen in Figure 3, across all conditions as their first action children showed a strong inclination to copy the majority, with this effect being strongest in the Positive condition (even though in this condition the lone individual's behavior was endorsed by the group) and weakest in the Frequency Control condition. In line with this, an ANOVA with condition as a between-participant factor was significant, *F* (3, 40) = 3.33, *p* = .029, partial η^2^ = .20. Tukey HSD post-hoc comparisons indicated that the mean number of actions produced by children in the Positive condition (*M* = 2.91; *SD* = .30) was higher than those in the Frequency Control condition (*M* = 2.18; *SD* = .87; *p* = .038). This difference between the Positive and Frequency Control condition is likely driven by a slightly higher proportion of children in the Frequency control condition opting for the lone model's action after seeing that demonstration three times. As the Positive condition had (marginally) the highest level of majority action copying, this was reflected in a significant difference between the Positive condition and the Frequency control. However, in pairwise follow-up tests, there was no significant difference between Negative reaction (*M* = 2.81; *SD* = .40) and the Positive condition (*p* = .985), or between the Neutral reaction control condition and the Positive condition (*M* = 2.45; *SD* = .69; *p* = .723), which would be indicative of an effect of the injunctive, over descriptive norm. Consistent with this interpretation, with regard to exhibiting the majority and minority actions at all, as is evident in Figure 3, there were no significant differences across conditions, *F* (3, 40) = 1.70, *p* = .182, partial η^2^ = .11 and *F* (3, 40) = 1.13, *p* = .348, partial η^2^ = .07 respectively. However, collapsed across conditions children were significantly more likely to produce the majority action at all (*M* = 2.75, *SD* = .49) than the minority action (*M* = .55, *SD* = .70), *t*(43) = 13.15, *p*<.001, *d* = 3.66.

Reflecting the low occurrence of the minority action, only 8 children switched from attempting the more difficult majority method to the easier minority method (7 children did the opposite, switching from the minority to the majority method). No child switched method on more than one apparatus and the tendency to do so was unaffected by condition, *F* (3, 40) = .09, *p* = .964, partial η^2^ = .01. Notably, children competently copied the actions shown to them (see Figure 3). That is, 32 of the 44 children tested opened all three apparatuses, with another 11 opening two, and one child opening one, a pattern unaffected by condition, *F* (3, 40) = 1.55, *p* = .218, partial η^2^ = .10.

## Discussion

The current experiment examined children's proclivity to copy alternative behaviors which were either descriptive norms (performed by the majority of individuals) or injunctive norms (a behavior receiving differing injunctive reactions by the group), within a design which mimics the observations of adult behavior which children receive during development. We found that the injunctive reaction of group members to a behavior had little impact on children's imitation. That is, children reacted by copying the actions demonstrated by the majority of group members, regardless of if the actions of the minority model were responded to by the observing group members positively, neutrally or negatively. This suggests that the children we tested had a strong majority bias, overcoming any tendency to adhere to the injunctive norms expressed by the group in the actions they used. Also, the relative efficiency and ease of the lone model's method of opening the apparatuses did not overcome children's preference for the slower and more time intensive majority actions.

Experimental evidence with adults (e.g., [28]), children (e.g., [39] [41]), chimpanzees [40], and fish [60] argue for the adaptive utility of copying the majority (for a discussion see [61]). Given group behavior typically develops out of combined individual learning efforts it is likely to be safer, more reliable and more productive to adopt than that of any lone response [34]. Moreover, as Chudek and Henrich [62] have argued, early cultural learners would have faced increasing selection pressures to adopt the majority practices of their community as coordination with community members came to represent an ever larger proportion of lifetime fitness.

The current results add to the growing body of evidence showing a majority bias in social learning: In a setting where both the attitudes and behaviors of the group are evident (i.e., their injunctive reactions, saliently underpinned by emotion, and their actions), children opt to imitate the behavior of the group rather than follow their disposition towards a behavior. This is perhaps not surprising from an evolutionarily perspective. Based on the logic that agents do not typically perform behaviors which are detrimental to themselves [34], the developing human should copy the agent's behavior because it is likely to be (a) safe, and (b) possibly advantageous. The attitudes of the group may reflect adaptive behavior in a less reliable manner.

It remains possible that children's responses were being driven by other processes that have little to do with normative or conformist behavior. For example, adopting the group action because it took longer to demonstrate. We are unaware of any evidence to suggest children will prioritize copying longer or more complicated sequences over shorter or less complicated ones (see [63]). While the recent research of Haun and colleagues [40], shows both human children and even chimpanzees pay special attention and copy specifically the action of the majority of individuals, which further makes alternative explanations based on presentation order or length unlikely. Congruently, Nielsen and Blank [53] presented children with two models who demonstrated different actions on the same apparatus. They reported that children would copy the actions of whichever model remained with them when they were given the apparatus to explore. It was found that the social context was key in driving children's behavior, and that the order in which the actions were modeled had no impact.

It is interesting to examine the current results in light of work into the theory of planned behavior. In the theory of planned behavior [50] evidence has tended to suggest that injunctive norms (what the group thinks you should do) more strongly predict intentions to perform behaviors than descriptive norms (what the group does; [35] [57] [64]). For example, Smith and Louis [57] conducted two studies that examined the interactive effects of injunctive and descriptive norms. They found injunctive norms to be a more consistent predictor of attitudes. Thus while work with adults has suggested a preference to perform behaviors in line with the attitudes of the group over the majority behavior, we found children prefer the opposite.

There are several possible explanations for this apparent but potentially informative discrepancy. Firstly, it is possible that the difference in results is traceable to differences in method. Smith and Louis [57] rely on self-report, whereas our experiment measured actual behavior. Smith and Louis used written explicit attitudes of the group, whereas we used behavioral and verbal emotional reactions. Yet it is also possible that our finding represents a pertinent developmental difference in the use of social information between children and adults. Children, being more vulnerable, may have a bias to use the more reliable and safer majority-behavior channel, later switching, as development proceeds, to the emotional valence and injunctive reaction of the group as the preferred source of information. This presents itself as a topic for future research.

We would not want to suggest on the basis of the current experiment that the emotional channel, injunctive reactions or attitudes expressed by adults are in general weak or that, under other circumstances, they would not be preferred. For instance, the emotional reactions of primary caregivers may be a highly weighted source of information. Furthermore, it is possible that emotional reactions directed at the child may cause a shift in their preferences. It is possible that children's disposition towards a behavior may be changed without their tendency to adopt that behavior being changed. Future research is needed to examine such possibilities.

There are inherent complexities in manipulating descriptive and injunctive norms within the same design to quantify their respective impact (e.g., [6]), as what most do and what they think you should do intrinsically interact, leading to issues which are hard to avoid in any one design. Take for example a manipulation where group members perform behaviors that they show disapproval towards, leading to a conflict of injunctive and descriptive norms that is not reflective of most real-world social interaction. We must further highlight the limitations which grow out of the inherent interactions of descriptive and injunctive norm (especially in research with children), how the current experiment sort to minimize the impact of these, and acknowledge the weakness still left in the design. Unlike in research with adults where injunctive and descriptive norms can be manipulated in subtle and less problematic ways, either in vignettes within survey research (e.g., [57]) or having participants make inferences from the situation (e.g., [7]), with children it is necessary to show the actual social interactions, as has been the precedent in previous majority influence research (e.g., [36]). It is also much more difficult to measure children's evaluations or dispositions, towards certain behaviors; where in adults you can attain ratings of their relative evaluation of behavioral options through self-report, with children a more common strategy is to measure behavior, inferring that this represents the source of information they are prioritizing (as is ubiquitous in imitation research). These methodological constraints combine to create a situation in which (at least) two behavioral options must be available from which you can infer which type of normative information children are prioritizing in response to a demonstration by a group, by which action children perform.

There are several apparent options in doing this. In the current experiment we chose a procedure in which a group of individuals was both performing a one set of actions, and displaying an injunctive reaction to a further individual, who was performing a second set of actions. This allowed us to infer the effect of the injunctive norm by examining the rate at which the actions of the lone individual were employed by children, compared to the group's actions. This design was selected for external validity, having the virtue of establishing one group who both establishes the descriptive norm, and provides injunctive reactions towards the actions of another individual. This is close to the situation children face in development where their group members will perform actions, and display reactions towards actions of individuals which differ from most of the groups.

Yet this design generates a complexity from the interaction of descriptive and injunctive norms. When the group shows a negative reaction towards the lone individual descriptive and injunctive norms are congruent: the group performs one action, and views negatively the use of another action. When the group shows a positive reaction towards the lone individual descriptive and injunctive norms conflict: the group performs one behavior, but looks on the alternative set of actions performed by the lone model as positive. This is more reflective of real social interaction than the converse (where the group derides a behavior they themselves perform), as there are many situations in which individuals who perform behaviors different from the majority of the group receive group approval: such as when an innovation is found, when an individual performs exceptionally (as in sport), or when certain roles dictate that only some group members can perform certain actions. But even in everyday life this type of conflict occurs, Individual's might approve of throwing rubbish in the garbage, even though they litter; or approve of not smoking even though they smoke.

An issue with this procedure of manipulating injunctive and descriptive norms with children, when only behavior is measured, is that it leaves opaque the degree to which children positively or negatively evaluate the actions used. For instance, in the case where the group produces a positive reaction towards the lone individual, we cannot asses the degree to which children evaluate the group's actions as positive (because they have been performed by a majority), and the relative degree to which they evaluate the actions of the lone individual as positive (because the group has reacted positively towards it). This kind of psychological attribution would be commonly assessed by self-report in adults, a tool which is problematic for children this early in development. That is, while it is possible for individuals to evaluate both things positively, when limited to measuring actions we can only infer that the behavior children produced reflects a prioritization of one form of information over another. If children in our study did evaluate the actions of the lone model as positive, after it received a positive reaction, it is notable that they did not employ this evaluation in the form of any higher proportion of copying behavior of the lone model's behavior. It is an important avenue for future research to (a) establish methods to examine if children's appraisals differ in this way, and (b), to establish if stronger manipulations of injunctive reaction can be produced to shift children away from the preference to copy the majority, as found in this experiment.

Another promising way to manipulate injunctive and descriptive norms, given the methodological constraints outlined above, would be to have one group establish a descriptive norm, by performing one set of actions, and have a second group produce an injunctive reaction towards a lone model performing a second set of actions. While this would have the advantage of not creating a situation where they group is approving of a behavior they are not performing (or disapproving of a behavior they are performing), it introduces a further artificiality. Namely, how children will interpret these two groups: whether they will see them as two sub-groups or as two distinct groups which may have different norms altogether. This further highlights the need for further experiments to be run, and the difficulty in ruling-out all possible alternative interpretations within in anyone design, in investigating the interaction of descriptive and injunctive norms.

It is interesting to consider the current research in the light of a recent experiment by Herrmann, Legare, Harris, & Whitehouse [65]. They presented children with videos of models performing an imitation task. Children either saw two videos of separate models successively, performing the task identically, or a video of the same model twice. They also had further conditions in which children saw the two models performing the task synchronously (this was further manipulated with children either seeing this video twice or once). Before seeing these videos children listened to a comment by the experimenter designed to frame the experience of the child, either saying “she always does it his way” or “she always gets the pegs up” (the aim of the task). The object of the experiment was to examine if children would differentially perceive tasks as purely instrumental or conventional; where conventional tasks (like rituals) require exact copying of actions, and instrumental tasks only require achieving the same outcome. To this end, Herrmann and colleagues measured the imitation fidelity of children and their explanations for their behavior. They found that the framing drawing attention to the conventionality of the actions “she always gets the pegs up” and the viewing of two synchronous demonstrations of adults promoted high-fidelity and more conventional-based explanations about the actions children chose to perform. These results add a further layer of complexity to majority influence and conformity research, as they suggest simply witnessing the actions of multiple adults performing a behavior may change the child's interpretation of what their imitative goals are within the experiment. Designs such as the one employed here may find children copy the majority's actions more closely (having higher imitation fidelity), because they interpret these actions as conventional. This may be especially so in the current experiment, where the majority's actions contained irrelevant actions, perhaps earmarking them as conventional.

In conclusion, children's ability to use social information in directing their behavior is key to their survival and development. However, this social information comes in several forms that may vary in their reliability and in the contexts in which they are most adaptive (see [66] [67]). The current experiment shows, at least in terms of incidental observations of social interaction, children prefer to conform rather than base their behavior on the injunctive reactions of models. Furthermore by examining how two differing sources of information interact, the current experiment also represents a more ecologically valid experimental approach which will become increasingly necessary if our understanding of the basic forces which drive social learning is to advance.
