# Correction: Groups' Actions Trump Injunctive Reaction in an Incidental Observation by Young Children

**DOI:** 10.1371/journal.pone.0114789

**Published:** 2014-11-25

**Authors:** The PLOS ONE Staff

The images for Figure 2 and Figure 3 are incorrectly switched. The image that appears as Figure 2 should be Figure 3, and the image that appears as Figure 3 should be Figure 2. The figure legends appear in the correct order.

**Figure 2:**
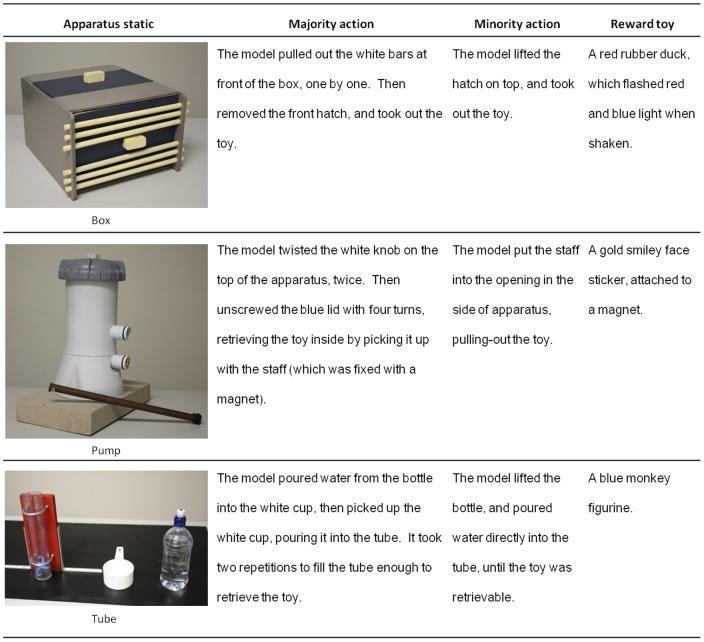
The three apparatuses used, descriptions of both sets of actions used to retrieve the reward toy, and description of the reward toys.

**Figure 3:**
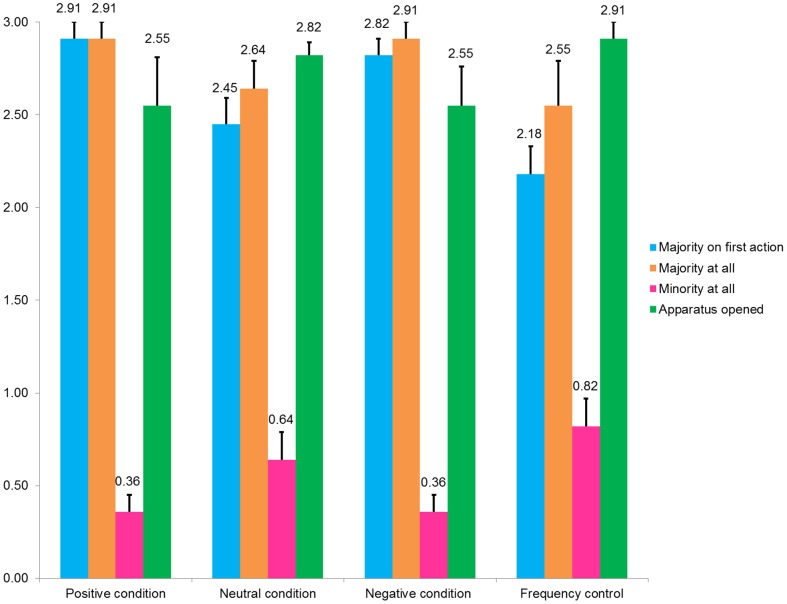
Means (and standard errors) across conditions for the number of apparatuses on key outcome measures.

## References

[1] Turner CR, Nielsen M, Collier-Baker E (2014) Groups' Actions Trump Injunctive Reaction in an Incidental Observation by Young Children. PLoS ONE 9(9): e107375 doi:10.1371/journal.pone.0107375 25198163 10.1371/journal.pone.0107375 PMC4157860

